# Dr. Isaac T. Mullen

**Published:** 1918-07

**Authors:** 


**Dr. Isaac T. Mullen** died in Chicago May 10th. He had lived formerly in Oakfield, N. Y.

